# Supplementary material for: Deletion of wfs1 Impairs Oligodendrocyte Precursor Cells Dorsal Distribution and Myelination Through the wfs1-hmgcs1 Axis in Zebrafish
Source: Biology (Basel). 2026 Aug 16;15(16):1402. doi: 10.3390/biology15161402 (PMC13510151; doi:10.3390/biology15161402)
Supplement: Supplementary file 1 [file biology-15-01402-s001.zip › File S1-Transcriptome sequencing data and differentially expressed genes (DEGs) in wfs1-- zebrafish at 5 dpf/2.KEGG/wfs1KOvsWT/ALL/src/dre04625.html]

dre04625


- dre:566185

- Up regulated genes

566185(6.72123660882883)

- dre:561021

- Up regulated genes

561021(2.14284154145004)

- Down regulated genes

561021\_1(-4.68369619674599)

- dre:436718

- Up regulated genes

436718(1.34229427553561)

- Down regulated genes

436718\_1(-6.85571323128554)

- dre:368481

- Down regulated genes

368481(-2.13889418398421)

- dre:368481

- Down regulated genes

368481(-2.13889418398421)

- dre:571242

- Down regulated genes

571242\_2(-1.95033382416063)

- dre:565241

- Up regulated genes

565241(1.32977867526604)

- Down regulated genes

565241\_2(-6.09874532631292)

- dre:556277

- Up regulated genes

556277(2.56614816386233)

- dre:571242

- Down regulated genes

571242\_2(-1.95033382416063)

- dre:571242

- Down regulated genes

571242\_2(-1.95033382416063)

- dre:571242

- Down regulated genes

571242\_2(-1.95033382416063)

- dre:100000252

- Up regulated genes

100000252\_3(1.94434154854557) 100000252(1.09163460881879)

- Down regulated genes

100000252\_1(-1.7718817168434)

- dre:795414

- Down regulated genes

795414\_2(-7.95634124062123)

- dre:795414

- Down regulated genes

795414\_2(-7.95634124062123)

- dre:795414

- Down regulated genes

795414\_2(-7.95634124062123)

- dre:192322

- Up regulated genes

336121(1.42904963424916)

- dre:565241

- Up regulated genes

565241(1.32977867526604)

- Down regulated genes

565241\_2(-6.09874532631292)

- dre:192322

- Up regulated genes

336121(1.42904963424916)

- dre:559020

- Up regulated genes

559020(1.27287729194892)

- dre:368241

- Up regulated genes

368241(1.70664623870244)

- dre:562552

- Up regulated genes

562552(1.50682290794657)

Close
